# Evaluation of Exosomal Coding and Non-Coding RNA Signature in Obese Adolescents

**DOI:** 10.3390/ijms24010139

**Published:** 2022-12-21

**Authors:** Manuela Cabiati, Emioli Randazzo, Letizia Guiducci, Alessandra Falleni, Antonella Cecchettini, Valentina Casieri, Giovanni Federico, Silvia Del Ry

**Affiliations:** 1 Laboratory of Biochemistry and Molecular Biology, Institute of Clinical Physiology, CNR, 56124 Pisa, Italy; 2 Unit of Pediatric Endocrinology and Diabetes, Department of Clinical and Experimental Medicine, University of Pisa, 56126 Pisa, Italy; 3 Department of Experimental and Clinical Medicine, University of Pisa, 56126 Pisa, Italy; 4 Scuola Superiore Sant’Anna, Unit of Translational Critical Care Medicine, 56126 Pisa, Italy

**Keywords:** exosomes, miRNA, obesity, childhood, Real-Time PCR

## Abstract

Exosomes may contribute to the pathogenesis of obesity through their action as communication mediators. As we have previously demonstrated, in obese adolescents, some circulating miRNAs modified the C-type natriuretic peptide (*CNP*) expression and were associated with changes in metabolic functions. At present no data are available on miRNA transport by exosomes in this condition. To verify and compare the presence and the expression of *CNP/NPR-B/NPR-C*, and some miRNAs (*miR-33a-3p*/*miR-223-5p*/*miR-142-5p*/*miRNA-4454*/*miRNA-181a-5p*/*miRNA-199-5p*), in circulating exosomes obtained from the same cohort of obese (O, n = 22) and normal-weight adolescents (N, n = 22). For the first time, we observed that exosomes carried *CNP* and its specific receptors only randomly both in O and N, suggesting that exosomes are not important carriers for the *CNP* system. On the contrary, exosomal miRNAs resulted ubiquitously and differentially expressed in O and N. O showed a significant decrease (*p* < 0.01) in the expression of all miRNAs except for *miR-4454* and *miR-142-5p*. We have found significant correlations among miRNAs themselves and with some inflammatory/metabolic factors of obesity. These relationships may help in finding new biomarkers, allowing us to recognize, at an early stage, obese children and adolescents at high risk to develop the disease complications in adult life.

## 1. Introduction

It is a truth universally acknowledged that adolescent obesity represents one of the greatest contemporary public health issues. The chronic imbalance between caloric intake and energy consumption, determined by a complex interaction among genetic, epigenetic, and environmental factors, leads to the development of overweight and obesity [1]. This condition in childhood and adolescence promotes systemic inflammation, consequently increasing the likelihood of metabolic and cardiovascular complications in adulthood [2].

At present, anthropometric tools and indicators, biochemical parameters, and biomarkers are used to evaluate the nutritional status of the population [3]. In this regard, biomarkers, namely circulating measurable molecules assessing the stability or the degree of abnormality of a particular biological process, allowed us to detect or monitor health deterioration, and very frequently, nutritional alterations. Bearing this in mind, in a recent paper we introduced the concept that expanding our knowledge on the behavior of circulating miRNA profiles might contribute to an early identification of obese children at increased risk of developing cardiometabolic alterations later in life [4]. At the same time, basic and applied research on extracellular vesicles (EVs) and their cargo showed significant interest in their important role in intercellular communication in several diseases including obesity. Briefly, EVs may be subdivided size-wise into exosomes (30–120 nm), microvesicles (100 nm–1 μm), and apoptotic bodies (2–3 μm). They are released by cells into the extracellular space and their release and content respond to the conditions surrounding the cellular microenvironment [5]. This response appears to be so highly calibrated that it promises predictability of response in the event of alterations within the microenvironment nearby the cells, allowing us to detect and measure these anomalies. This could help to bridge the gap between deteriorating health and time to detection.

The use of EVs as potential biomarkers in some conditions, such as neurodegenerative, cardiovascular, chronic-degenerative diseases, and in some types of cancer [6,7,8,9], has been suggested. More recently, it has been reported that exosomes may also contribute to the pathogenesis of obesity and associated disorders through their action as communication mediators [10]. Exosomes have been isolated in various biological specimens, including blood, urine, semen, breast milk, saliva, amniotic fluid, cerebrospinal fluid, synovial fluid, and in broncho-alveolar lavage [11]. If exosome-mediated intra-adipose and inter-organ communication are of great significance for energy metabolism, then we should consider that this special function of exosomes in adipose tissue biology may be disrupted in obesity [12]. The aim of our study was to verify whether the exosomes isolated from plasma samples, obtained partly from a cohort of obese adolescents and partly from a group of healthy normal-weight subjects contained the mRNAs of *CNP*, *NPR-B*, *NPR-C*, and some miRNAs linked to obesity, and if so, how their expression levels behaved in the two groups. From this perspective, the study of exosome-mediated biomarker transfer constitutes a new approach; thus, the results of this analysis could provide important indications for future innovative pharmacological treatments.

## 2. Results

### 2.1. Clinical Characteristics and Biochemical Determination

As previously reported [4,13,14,15,16,17,18], O and N had different clinical characteristics (Table 1). We observed that O and N had similar circulating levels of fasting blood glucose with HbA1c levels that were significantly higher in O, but still in the normal range indicating that they had not developed overt Type 2 diabetes yet. The increased levels of HOMA-IR and circulating insulin, total and LDL cholesterol, and triglycerides showed that O adolescents had reduced insulin sensitivity together with a certain degree of dyslipidemia.

### 2.2. Characterization of Vesicles Isolated from Plasma

**Figure 1:**
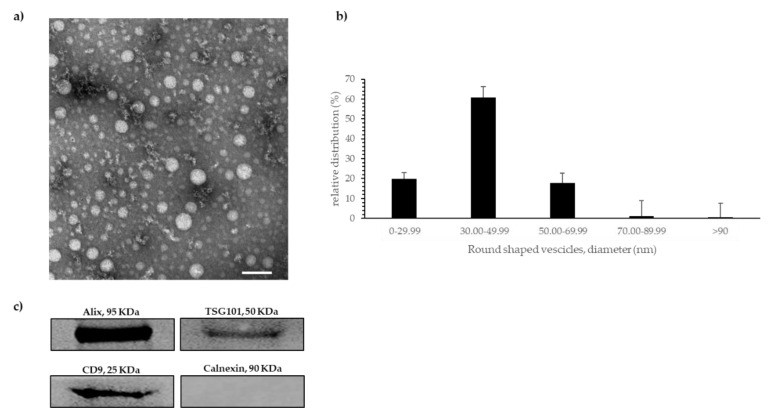
(**a**) Representative Transmission Electron Micrograph of round shaped vesicles. Scale bar = 100 nm. (**b**) Diagram shows the vesicles relative distribution in classes according to their dimensions. (**c**) Western blotting analysis of proteins obtained from the isolated exosomes. Bands show the recognition of the specific exosomal markers Alix, TSG101, and CD9, but not of Calnexin.

Following MISEV 2018 guideline [19] for exosome study, the exosome population was characterized with both TEM and Western blotting analyses. TEM analysis revealed that the exoRNeasy mini/midi kit procedure isolated round-shaped intact vesicles from pre-filtered plasma samples (Figure 1a).

Figure 1b shows the relative distribution of vesicles in classes according to their dimensions. Up to 60% of vesicles were in the 30–49.99 nm class, 20% in the 0–29.99 nm class, 19% in the 50–69.99 nm class. Wider EVs (ranging from 70 to >90 nm) were less represented (<10%).

Western blotting analysis detecting proteins specifically expressed in the exosomes, confirmed TEM results. In particular, Western blot identified proteins known to be part of the exosomal internal cargo, such as TSG101 and Alix, as well as a surface exosomal protein, for instance CD-9. As expected, Calnexin, a protein not expressed by exosomes, was not recognized (Figure 1c). 

Taken together, these results indicated that the procedure we followed allowed us to properly isolate and purify from plasma an enriched population of exosomes. The terms “EVs” and “exosome” we used in the text to describe our results mean “enriched population of exosomes”. These samples were used for Real-Time PCR analyses.

### 2.3. Real-Time PCR Data Analysis

Bearing in mind the results we obtained in our previous studies [4,13,14,15,16,17,18], we investigated whether biomarkers, such as *CNP* and its specific receptors (*NPR-B, NPR-C*), together with some *miRNAs* (*miR-33a-3p*, *miR-223-5p*, *miR-142-5p*, *miR-4454*, *miR-181a-5p* and *miR-199-5p*), were carried out by circulating exosomes and whether their transcriptional profile in obese and in normal-weight adolescents differed from one another. 

As far as *CNP* and its specific receptors are concerned, we observed that, in both obese and normal-weight individuals, their mRNAs were randomly present in the exosomes. 

**Figure 2:**
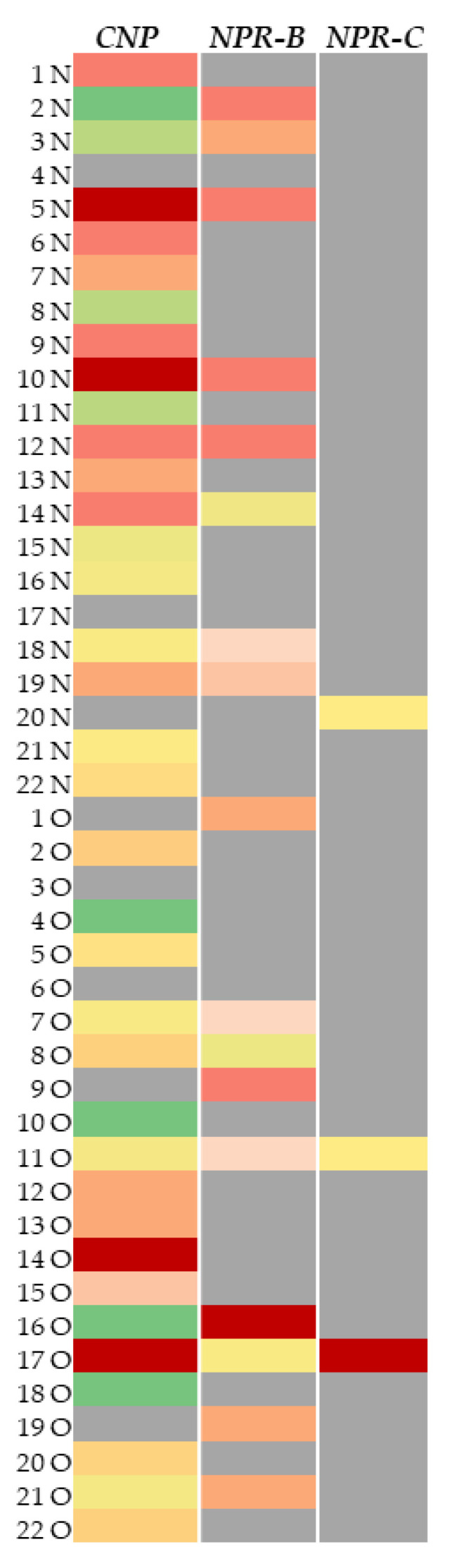
Heat-map representation of mRNA expression levels of *CNP*, *NPR-B*, and *NPR-C*. Dark red: higher expression levels (≥33 cycles), green: lower ones (>38.5 cycles), grey: No expression. Median levels of expression were represented in light red, light green, orange and yellow (35.5 < X < 38 cycles) (N = Normal-weigh subjects; O = Obese subjects).

The heat-map graph (Figure 2) shows with more clarity the expression trends of *CNP*, *NPR-B*, and *NPR-C* mRNAs. Individual results, expressed in terms of Ct, were visualized by as a scale of colors: dark red indicated a higher expression level, green a lower one, while grey indicated no expression at all. Median expression levels were represented in light red, light green, orange and yellow. Observing the heat-map graph, the *CNP* mRNA expression was uniform in O and N individuals. *NPR-B* and *NPR-C* were expressed only in a few samples (*NPR-B*:N, n = 8/O, n = 9; *NPR-C*:N, n = 2/O, n = 1) (Figure 2). 

**Figure 3:**
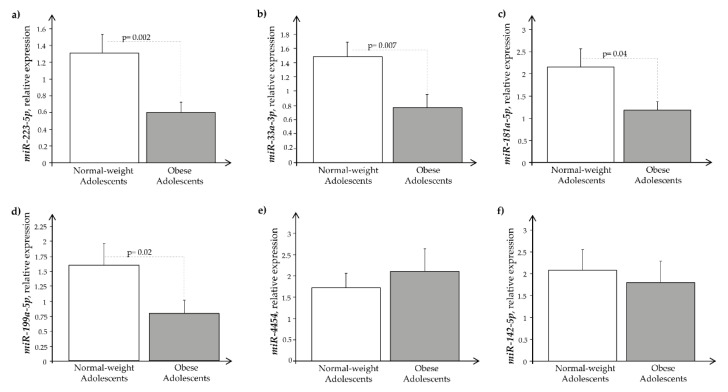
Expression level of exosomal miRNAs. (**a**) *miR-223-5p*, (**b**) *miR-33a-3p*, (**c**) *miRNA-181a-5p*, (**d**) *miRNA-199-5p*, (**e**) *miRNA-4454*, (**f**) *miR-142-5p* in normal-weight (open bars) and obese subjects (closed bars).

Regarding miRNAs, we observed that, in contrast to *CNP* and its specific receptors mRNAs, they were ubiquitously detected in our exosome samples, showing different expression patterns between O and N subjects. In particular, obesity had significantly reduced expression levels of *miR-223-5p*, *miR-33a-3p*, *miR-181a-5p*, and *miR-199-5p*, as displayed in Figure 3-(Figure 3a–d), while the remaining miRNAs, *miR-4454* and *miR-142-5p* (Figure 3e–f) showed only small, not significant, changes.

**Figure 4:**
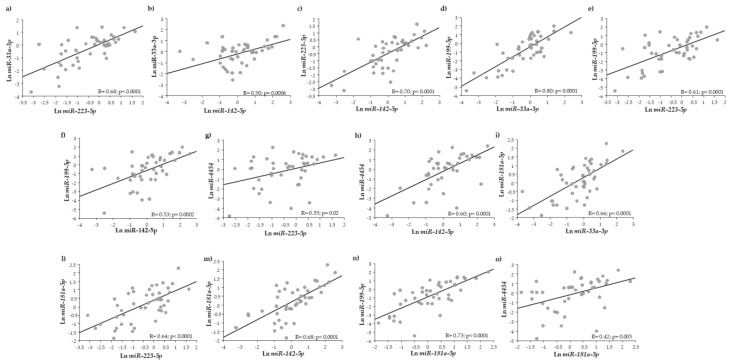
Correlations among exosomal miRNAs.

As reported in Figure 4, we found significant correlations among *miR-33a-3p* and *miR-223-5p*, *miR-142-5p* (Figure 4a,b, respectively); *miR-223-5p* and *miR-142-5p* (Figure 4c); *miR-199a-5p* and *miR-33a-3p*, *miR-223-5p*, *miR-142-5p* (Figure 4d–f, respectively); *miR-4454* and *miR-223-5p*, *miR-142-5p* (Figure 4g,h, respectively); *miR-181a-5p* and *33a-3p*, *miR-223-5p*, *miR-142-5p*, *miR-199a-5p*, *miR-4454* (Figure 4i,l–o, respectively). 

Interestingly, we observed that some miRNAs significantly and positively correlated with metabolic and inflammatory indices. In particular, *miR-142-5p* correlated with total cholesterol (R = 0.45; *p* = 0.008) and LDL (R = 0.43; *p* = 0.02), *miR-4454* with total cholesterol, HDL and LDL (R = 0.51; *p* = 0.004; R = 0.38; *p* = 0.03; R = 0.49; *p* = 0.008), and *miR-181a-5p* with LDL (R = 0.38; *p* = 0.03). PCR correlated significantly with *miR-223-5p* (R = 0.33; *p* = 0.05) and *miR-199-5p* (R = 0.41; *p* = 0.01). 

Including both obese and normal-weight subjects as dependent variables and miR-*181a-5p*, *miR-199a-5p*, *miR-223-5p*, *miR-33a-3p*, and *miR-4454* together with auxologic parameters as predictors in a multivariate logistic regression model, we observed that miRNAs were not significant predictors of childhood obesity (Table 2a). However, by replacing auxologic parameters with some metabolic indices in the regression model, as independent variables, we found that *miR-4454*, and HDL (Table 2b) were independent predictors of childhood obesity (*miR-223-5p* was only marginally significant). 

Employing miRWalk (http://mirwalk.umm.uni-heidelberg.de/) [20], an open-source platform for miRNA binding site, we found interactions between *miR-4454*, *miR-223-5p*, *miR-142-5p*, and *miR-181a-5p* and some target genes involved in the regulation of lipid metabolism.

**Figure 5:**
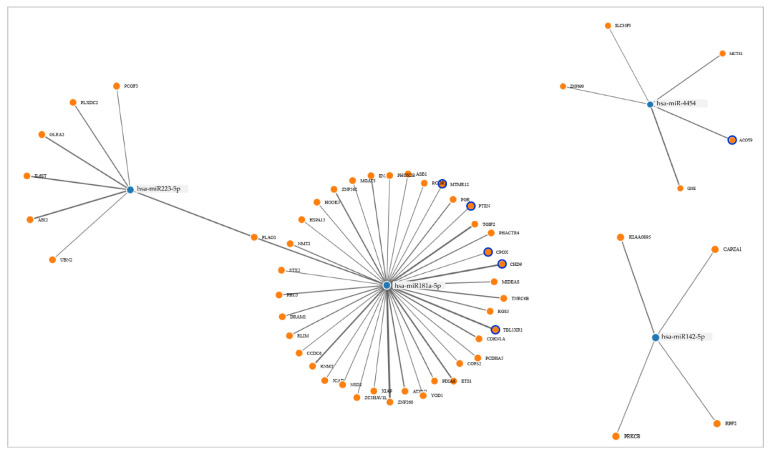
Network graph plot. The miRNA-target gene interactions are displayed as a node graph. Blue rings show miRNA–target genes associated with lipid metabolism.

These miRNAs were further analyzed by the Gene Set Enrichment Analysis (GSEA) that revealed their interaction with 54 target genes. Applying the Kegg pathways algorithm to these genes, they were split into cancer and metabolic pathways. Thereafter, using a database providing bioinformatics tools for pathways visualization, interpretation, and analysis (Reactome, https://reactome.org/) [21], we extrapolated the genes most linked to the metabolic pathway that were those linked to lipid metabolism (Chromodomain Helicase DNA Binding Protein 9, *CHD9*; Phosphatase and tensin homolog, *PTEN*; Myotubularin Related Protein 12, *MTMR12*; TBL1X/Y Related 1, *TBL1XR1*; Coproporphyrinogen Oxidase, *CPOX*; Acyl-CoA Thioesterase 9, *ACOT*9). The network graph plot (Figure 5) shows that *miR-223-5p* interacts with *miR-181a-5p* and that *miR-4454* and *miR-181a-5p* are closely linked to genes involved in lipid metabolism, reinforcing our finding, shown by linear regression analysis, of a relationship between the expression levels of these miRNAs in the exosomes and the circulating levels of lipids.

## 3. Discussion

Exosomes secreted by plasma membranes may act directly with cell surface receptors by interacting with their transmembrane proteins in two ways: Via lipid ligands or by releasing their cargo into recipient cells through a membrane fusion process.

Their biological effects may, therefore, involve both nearby and remote target cells. Starting from these considerations, it is not surprising that exosomes may have significant roles in a variety of diseases. In this regard, it has been reported that patients with metabolic dysfunction had increased circulating amounts of exosomes [22]. Moreover, it has also been observed that obesity and other inflammatory conditions were associated with an increased number of vesicles [23], indicating that the number of exosomes can be a marker of these diseases and that serum or plasma-derived exosomes are an interesting field of research for finding new biomarkers. Exosomes, in fact, contain many bioactive substances, including miRNAs, involved in the regulation of fundamental cell functions such as growth, differentiation, and metabolism. In addition, it is interesting to note that miRNAs carried into exosomes are sheltered from degradation induced by RNase contained in body fluids, suggesting that, in the future, exosomes could be used as carriers of miRNAs, employed in medical therapies.

In a previous study, we evaluated the expression levels of some biomarkers in obese and normal weight adolescents using blood RNA samples [4] and we found that some miRNAs were independent determinants of circulating *CNP* levels. In the present study, we investigated, the expression levels of the same biomarkers in the same individuals, this time using RNA extracted from exosomes isolated from plasma: while the results on the expression of *CNP* and its specific receptors were disappointing to a certain extent, those obtained on the expression of miRNAs were instead quite interesting. As far as the mRNAs of *CNP*, *NPR-B*, and *NPR-C* were concerned, in fact, those are the first data about their transport by exosomes [10,23,24,25,26,27]; however, we found that exosomes carried only randomly these mRNAs both in O and in N. These results on the expression of the *CNP* system in the exosomes suggest that exosomes are not important carriers for *CNP* and therefore not helpful as biomarkers and for specific therapeutic purposes. 

Regarding exosome miRNAs, we observed, to our knowledge for the first time, that they were ubiquitously and differentially expressed in samples obtained from O and N adolescents. 

It is interesting to underline that, while our previous data [4] on the expression levels of the same circulating miRNAs showed higher levels in obese than in normal-weight adolescents, the results we obtained in the circulating exosomes indicated a reversed behavior, with lower expression levels in obese than in normal-weight adolescents. It is not easy to explain why circulating and exosome miRNAs behave so differently; however, some considerations can be drawn. 

One explanation may be that circulating and exosome miRNAs reflect the results of the regulation of these biomarkers in two different compartments, that is plasma and cell-derived exosomes. In particular, the plasma trend of miRNAs not specifically recapitulates biological mechanisms deriving from several districts (cells, tissues, etc.), while the expression of exosome miRNAs may reflect the result of a more refined regulation [28]. The special role that exosomes and their cargo of non-coding RNAs (primary miRNAs) play in intercellular communication is known [24]. It is also important to remember that exosome biogenesis and secretion are influenced by the metabolic status of the cell which, in turn, depends on factors such as ceramide metabolism, endoplasmic reticulum stress, autophagy, and intracellular calcium [25]. After their release, exosomes interact with their target cells by transferring their bioactive cargo into them. As a result, this transfer triggers phenotypic changes in the recipient cells [10]. Taken together, these considerations may contribute to explain, at least in part, the different results we found in investigating the expression behavior of the same miRNAs in plasma and in exosome samples.

As reported above, exosomes and their cargo are altered in obesity [24] and there is evidence that they contribute to obesity and its cardiometabolic sequelae by acting as signaling entities [27]. This implies that their biological properties could have great potential in the diagnosis and treatment of obesity and its complications. In addition, since exosome-mediated intra-adipose and inter-organ communication is of great significance for energy metabolism, an explanation could be that this special function of exosomes in adipose tissue biology may be disrupted in obesity [10]. This could explain our findings of lower expression levels of *miR-223-5p* and *miR-33a-3p* in the exosomes of O in comparison to those of N adolescents. These two biomarkers are, in fact, known to be regulators of lipid metabolism [29,30,31,32,33,34].

In particular, *miR-223-5p* plays a critical role in systemic cholesterol and lipoprotein metabolism [29] and in the regulation of both inflammatory response and insulin resistance [30,31,32]. Moreover, *miR-33a-3p* is also a central regulator of lipid metabolism, vascular homeostasis and cardiac adaptation in response to pressure overload [33,34]. The results we obtained by linear regression analysis in our obese adolescents confirmed the association between *miR-4454*, *miR-223-5p*, *miR-142-5p* and *miR-181a-5p* and some metabolic factors in obese subjects [35,36]. Bioinformatic analysis reinforced these data, allowing us to extrapolate some genes involved in lipid regulation and closely linked to these miRNAs, highlighting their possible involvement in the mechanisms underlying the metabolic syndrome. In addition to the classic regulators of metabolic homeostasis, recent evidence showed that small non-coding RNAs have a remarkable role in the post-transcriptional regulation of a number of genes and that they are involved in many diseases [37], as also suggested by our results in childhood obesity.

## 4. Materials and Methods

### 4.1. Subjects and Plasma Collection 

The investigation conforms to the principles outlined in the Declaration of Helsinki (Br Med J 1964; ii:177). The study was approved by the local Ethics Committee and informed consent was obtained from the parents of each subject or from the subject himself/herself, as appropriate. Exosomes were obtained from blood samples of obese (O, n = 22) and normal-weight (N, n = 22) subjects collected during our previous studies [4,13,14,15,16,17,18] and whose clinical characteristics and biochemical variables are summarized in Table 1. Obese adolescents were referred to as outpatients to the Unit of Pediatric Endocrinology and Diabetes, Department of Clinical and Experimental Medicine, University of Pisa, Italy. As previously reported in our studies [4,13,14,15,16,17,18], we enrolled subjects with primary obesity, not induced by drug assumption or by disease, and not affected by diabetes or by cardiac dysfunction. Obesity was defined according to the International Task Force on Obesity in childhood criteria using population reference data specific for age and sex for body mass index (BMI) [38]. Normal-weight adolescents were healthy subjects, who repeated blood examination after an intervening disease. At the time of blood sampling, they were not assuming drugs from at least one week and blood results, including indices of inflammation, were in the normal range. BMI was calculated using the formula (weight (kg)/height m^2^) [39]. We used the same National reference data [38] to calculate BMI z-score and Height z-score. Total body fat (%) was measured using the Tanita BC-418 Segmental Body Composition Analyser (Tanita Corporation, Tokyo, Japan) [40]. Blood pressure was measured by trained investigators according to a standardized protocol [41]. 

We collected blood samples from all the subjects by venipuncture, performed in the morning after overnight fasting. To evaluate the circulating levels of biochemical markers, blood samples were collected into EDTA (1 mg/mL) and lithium-heparin containing vials [4,13,14,15,16,17,18]. Biochemical parameters reported in Table 1 were measured by appropriate commercial kits as previously reported and HbA1c was assessed by HPLC [4,13,14,15,16,17,18]. HOMA-IR (HOmeostasis Model Assessment of Insulin Resistance) index was calculated according the formula: Fasting plasma insulin in μU/mL × FPG in mmol/L/22.5 [42,43].

### 4.2. Exosomes Isolation and Vesicular RNA Extraction

Exosomes were extracted from 600 μL of plasma using a dedicated and innovative assay (exoRNeasy mini/midi kit, QIAGEN GmbH, Hilden, Germany), designed for rapid purification of total vesicular RNA, including noncoding RNA, mRNA, miRNAs, and other small RNAs, and performed according to the manufacturer’s protocol. The assay uses a membrane-based affinity binding step to isolate exosomes and other EVs from cell-free biofluids that result in only a low percentage with respect to exosomes.

Briefly, plasma was prefiltered using syringe filters excluding particles larger than 0.8 µm (Millipore® Membrane Filter, 0.8 µm pore size, MILLEX-AA, Merck, D), then exosomes were isolated adding in a 1:1 ratio a 2× binding buffer (XBP). Next, samples were transferred into the ExoEasy membrane affinity column, to bind the exosomes to the membrane, and then centrifuged at 500× *g* for 2 min at RT. After discarding the flow through, 3.5 mL of wash buffer (XWP) was added to the column, according to the starting plasma quantity, and centrifuged at 5000× *g* for 5 min at RT. Particulate matter other than vesicles, such as larger protein complexes or lipids, was largely removed during the binding step and the ensuing wash step. Vesicles were lysed by adding a phenol/guanidine-based combined lysis reagent (QIAzol) to the spin column, and the lysate was collected by serial centrifugations. 

After lysis, elution step and addition of chloroform, the resulting sample was separated into aqueous and organic phases by centrifugation. The aqueous phase was transferred to a new tube, and ethanol was added to provide appropriate binding conditions for all RNA molecules, including miRNAs and other small RNAs. 

Samples were then applied to the RNeasy MinElute spin column allowing to bind to the membrane. The column was washed once with buffer RWT, and then twice with buffer RPE to efficiently remove phenol and other contaminants. Finally, high-quality exosomal RNA was eluted in a small volume of RNase-free water (16 μL) and stored at −80 °C. RNA integrity, purity and concentration, were assessed by measuring absorbance at 230, 260, and 280 nm (NanoDrop Thermofisher, Waltham, MA, USA) and applying the Beer-Lambert law (expected values between 1.8–2.1, for protein contamination). The mean RNA concentration for obese and normal-weight adolescents was: O = 24.0 ± 2.5 ng/μL and N = 24.4 ± 1.9 ng/μL.

### 4.3. Exosome Characterization

To characterize circulating exosomes, we collected a plasma sample from two healthy volunteers, and after exosomes were isolated and before RNA extraction, we analyzed their morphology with TEM and we looked for the presence of specific exosomal proteins in our preparations by using Western blotting analysis. 

Transmission Electron Microscopy (TEM): Samples of vesicle suspension were obtained by adding 2 mL of XE buffer directly to the column before total exosomal RNA extraction. Samples were then prepared according to the negative staining procedure. In brief, 10 μL of the sample containing vesicles were fixed in 2% paraformaldehyde in 0.1 M cacodylate buffer and placed onto a 200-mesh formvar/carbon copper grids and allowed to settle for 3 min at RT. Grids were then washed with distilled water and 20 μL of an aqueous solution of uranyl acetate (2% *w/v*) was applied removing the excess with filter paper after 30 sec. The uranyl acetate solution was filtered through a 0.45 μm polycarbonate filter to remove any impurities before their deposition. Finally, the grids were air-dried for 15 min and observed using a Jeol 100SX TEM operating at 80 kV.

Four grids were examined and 34 micrographs at 20,000–40,000 X direct magnification were obtained with an ATMxR80b Camera System. The diameter of the vesicles was determined using the Image J software. A total of 615 diameters were measured. Moreover, following MISEV guidelines [19], we have submitted all relevant data of our experiments to the EV-TRACK knowledge base (EV-TRACK ID: EV220408) [44]. 

Western blotting analysis: Samples of vesicle suspension were obtained by adding 50 μL of cold RIPA buffer, containing protease and phosphatase inhibitors (Pierce, Rockford, IL, USA), which is able to lysate the exosomes, directly to the ExoEasy membrane column. Protein concentration was determined using BCA protein assay kit (Pierce, Rockford, IL, USA). Equal amounts of protein were fractionated by 10%–12% SDS polyacrylamide gel and transferred to nitrocellulose membrane (Bio-Rad Laboratories Inc., Hercules, CA, USA). Equal loading was controlled by Ponceau staining. Membranes were blocked with 5% nonfat dried milk in TBS/Tween20 (0.01%) at room temperature for 1 h, and then probed with primary antibodies at a predetermined concentration, at 4 °C, overnight.

Primary antibodies were used to detect: human CD9 (monoclonal antibody, 1:1000, #SA35-08, Novus Biological, San Diego, CO, USA), human TGS101 (polyclonal antibody, 1:1000; #T5701, Sigma-Aldrich Chemical, St. Louis, MO, USA), human Alix (monoclonal antibody, 1:1000, #2171, Cell Signaling Technology, Boston, MA, USA), which are established exosomal markers and human Calnexin (polyclonal antibody, 1:1000, ab10286, Abcam, Cambridge, UK), as negative exosomal marker. After incubation with these primary antibodies, and rinsing with TBS/Tween20 (0.01%) 3 times for 10 min, membranes were incubated with appropriate horseradish peroxidase-conjugated (HRP-conjugated) anti-rabbit (#A0545) and anti-mouse (#A9044) secondary antibodies (Sigma-Aldrich Chemical, St. Louis, MO, USA) for 1 h at room temperature. After incubation with specific secondary antibodies, membranes were washed 3 times for 10 min with TBS/Tween20 (0.01%). Specific protein bands were detected using ECL Plus Western Blotting Detection System (Pierce, Rockford, IL, USA) and western blot markers and protein imaging acquisition was performed by using the UVITEC Cambridge Imaging System (UVITEC, Cambridge, UK). Densitometric analysis of protein bands was carried out with Image J software (National Institute of Health, Bethesda, MD, USA).

### 4.4. Reverse Transcription and Real-Time PCR

The exosomal RNA reverse transcription was carried out with a dedicated kit for the simultaneous detection of total RNA and miRNA (miScript ® II RT kit, Qiagen, Hilden, Germany) in a thermal cycler (MyCycler, Bio-Rad Laboratories Inc., Hercules, CA, USA): 60 min at 37 °C, 5 min at 95 °C and 4 °C for ∞. cDNA samples were conserved, as appropriate, and diluted at +4 °C. Real-Time PCR analysis was performed in duplicate in the Bio-Rad C1000™ thermal cycler (CFX-96 Real-Time PCR detection systems, Bio-Rad Laboratories Inc., Hercules, CA, USA) and cDNA amplification reactions were monitored with a fluorogenic DNA binding dye (SsoFAS EvaGreen Supermix Bio-Rad Laboratories Inc., Hercules, CA, USA). Since Real-Time PCR efficiency is highly dependent on the primers used, their sequences were accurately designed with a specific software (Beacon Designer®; Premier Biosoft International, Palo Alto, CA, USA), while the mature miRNA sequences used as forward primer for miRNAs detection were downloaded from the miRBase database (www.mirbase.org) (Table 3). All the primers were synthesized by Sigma Aldrich (Milan, Italy). 

The *C. elegans miR-39 miRNA* mimic, used to normalized miRNA data, was measured by a custom standardized assay (miScript Primer Assay). As internal control, we analysed the expression of RNAY4 (Ro60-Associated Y4), a conserved RNA involved in sorting RNAs into exosomes ad EV as well.

To assess product specificity, amplicons were systematically checked by melting curve analysis, which was generated from 65 °C to 95 °C with increments of 0.5 °C/cycle. All experiments followed the MIQE (Minimum Information for publication of Quantitative Real-Time PCR Experiments) guidelines [45].

### 4.5. Statistics

Statistical analysis was performed using Statview 5.0.1 Software for Windows (SAS Institute, Inc., Cary, NC, USA). 

Relative quantification was performed by the ΔΔCt method using Bio-Rad’s CFX96 manager software (CFX-96 Real-Time PCR detection systems, Bio-Rad Laboratories Inc., Hercules, CA, USA).

Skewed variables were log-transformed before statistical analysis. Differences between more than two independent groups were analyzed by Fisher’s test after ANOVA, while relations between variables were assessed by linear or multivariate logistic regression analysis. The results were expressed as mean ± S.E.M. and a *p*-value < 0.05 was considered significant.

A heat map of the *CNP*, *NPR-B*, and *NPRC* expression data was also generated by a specific tool that combines Maestro Manager software (Bio-RAD) and excel to create a scale of colors indicating the gene expression in terms of cycle numbers (Ct).

Potential miRNAs and target genes interactions were predicted using the miRWalk database [19] which includes a machine-learning algorithm based on experimentally verified miRNA-target interactions. The Reactome database was also used to better analyze the pathways [20].

## 5. Conclusions

Exosomes characterize a novel, skilled, class of microvesicles with possible applications both as biomarkers in various illnesses and beneficial biomolecule carriers [24]. Due to their ability to transport nucleic acids and to target specific cells through their surface proteins, they can work as therapeutic delivery systems for miRNA mimetics or anti-miRNA oligonucleotides.

Several papers, including those we had analyzed, reported data on circulating miRNA, in obese children and adolescents [10,29,30,31,32,33], but studies on exosomal miRNAs in this condition are still few, as stated in a recent review [10]. We have demonstrated with this study, carried out for the first time on adolescents with obesity, that their plasma contained exosomes carrying some miRNAs whose expression levels behaved differently from those observed in normal-weight adolescents. The close relationship between some of these biomarkers and indices linked to inflammation and metabolic complications of obesity is of great interest considering that, if confirmed on larger studies, our data may help in finding new biomarkers allowing clinicians to recognize at an early stage obese children and adolescents at high risk to develop the disease complications in adult life. Furthermore, considering the peculiar carrier activity of exosomes and their sheltering ability against miRNA degradation, the identification of specific miRNAs involved in obesity might have, therapeutical applications in the future considering the peculiar carrier activity of exosomes and their sheltering ability against miRNA degradation.

Moreover, as far as the use of circulating miRNAs as biomarkers is concerned, we must take into account that human blood contains a variety of cell types, making it challenging to identify the cell origin of the particular miRNA analyzed. As reported in a recent review [46], the debate on the preference and recommendation of the source of miRNAs, whether exosomal or non-exosomal, is everlasting, highlighting that exosomal miRNAs can act as a better source for biomarker studies due to their advantages in terms of quantity, quality, and stability. In this regard, the exosomal source of miRNAs seems to hold promise in studies related to biomarkers and diseases, such as childhood obesity. Indeed, miRNAs from exosomes could be helpful in the screening and surveillance, thus allowing more efficient and prompter prevention, diagnosis and treatment of diseases.

## Figures and Tables

**Table 1:** Demography, clinical characteristics, body composition and biochemical variables of the study population.

	Normal-Weight Adolescents	Obese Adolescents	*p*
Age (years)	13.1 ± 0.2	12.6 ± 0.4	ns
Male: Female	12:10	19:13	ns
Height (cm)	158.2 ± 1.1	156.3 ± 2.0	ns
Height z-score	0.3 ± 0.08	0.6 ± 0.2	ns
Weight (kg)	53.3 ± 2.5	72.7 ± 2.9	*<0.0001*
BMI	21.1 ± 0.6	29.5 ± 0.8	*<0.0001*
BMI z-score	0.9 ± 0.1	2.7 ± 0.1	*<0.0001*
Fat Mass (kg)	12.2 ± 1.7	26.9 ± 2.3	*<0.0001*
SBP (mmHg)	113.2 ± 1.4	112.6 ± 2.1	ns
DBP (mmHg)	61.7 ± 1.2	65.7 ± 1.7	ns
FBG (mmol/L)	4.8 ± 0.1	4.4 ± 0.1	*0.02*
HbA1c (%)	5.2 ± 0.07	5.4 ± 0.06	*0.04*
Insulin (μU/mL)	8.1 ± 0.8	19.4 ± 2.5	*0.0002*
HOMA-IR	1.0 ± 0.09	2.4 ± 0.3	*0.0003*
HDL-C (mg/dL)	46.7 ± 2.7	45.7 ± 2.2	ns
LDL-C (mg/dL)	82.3 ± 6.9	109.7 ± 6.6	*0.007*
TC (mg/dL)	145.4 ± 8.4	167.7 ± 7.4	*0.05*
TG (mg/dL)	81.5 ± 17.7	88.6 ± 8.7	ns

**Table Legend.** All data are expressed as mean ± SEM. **BMI:** body mass index; SBP: Systolic Blood Pressure; DBP: Diastolic Blood Pressure; **FBG:** Fasting Blood Glucose; **HbA1c:** glycated hemoglobin; **HOMA-IR:** HOmeostatic Model Assessment of Insulin Resistance; **HDL-C:** High-Density Lipoprotein Cholesterol; **LDL-C:** Low-Density Lipoprotein Cholesterol; **TC:** Total Cholesterol; **TG:** Triglycerides.

**Table 2:** **(a)** independent determinants of obesity (Response Variable: Auxologic Parameters); **(b)** independent determinants of obesity (Response Variable: Metabolic Indices).

(a)	B (SE)	t	*p*
INTERCEPT	−14.44 (6.87)	−2.10	0.05
*miR-33a-3p*	0.008 (0.13)	0.06	0.95
*miR-223-5p*	−0.16 (0.13)	−1.28	0.22
*miR-142-5p*	0.08 (0.11)	0.73	0.47
*miR-199a-5p*	−0.05 (0.09)	−0.57	0.58
*miR-4454*	0.01 (0.05)	0.19	0.85
*miR-181a-5p*	−0.09 (0.18)	−0.48	0.64
Age	−0.02 (0.17)	−0.14	0.89
Weight	−0.09 (0.05)	−1.86	0.08
Height	−0.11 (0.05)	−2.24	*0.03*
Height z-score	−0.14 (0.23)	−0.60	0.56
BMI	0.11 (0.14)	0.74	0.48
BMI z-score	0.80 (0.29)	2.78	*0.01*
Fat Mass	0.005 (0.01)	0.36	0.72
Lean Mass	−0.01 (0.009)	−1.63	0.12
**(b)**			
INTERCEPT	0.75 (1.42)	0.52	0.61
*miR-33a-3p*	0.31 (0.19)	1.59	0.14
*miR-223-5p*	−0.43 (0.19)	−2.21	*0.05*
*miR-142-5p*	0.15 (0.15)	1.02	0.33
*miR-199a-5p*	−0.04 (0.12)	−0.38	0.71
*miR-4454*	0.21 (0.08)	2.63	*0.02*
*miR-181a-5p*	−0.09 (0.18)	−0.48	0.64
Total cholesterol	0.05 (0.03)	1.57	0.15
Triglycerides	−0.01 (0.007)	−2.01	0.07
HDL	−0.83 (0.04)	-2.32	*0.04*
LDL	−0.05 (0.03)	−3.29	0.17
HbA1c	0.16 (0.26)	0.61	0.55
HOMA-IR	0.23 (0.12)	1.86	0.09

Data were Log transformed before analysis when necessary.

**Table 3:** Mature miRNA sequence and gene oligonucleotides.

Gene	Forward Primer Sequence (5’---3’)	GenBank, Accession Number	Location	Ta, °C
***hsa-miR-33a-3p***	CAATGTTTCCACAGTGCATCAC	NR_029507	chr 22q13.2	55
***hsa-miR-223-5p***	CGTGTATTTGACAAGCTGAGTT	LM608368	chr Xq12	55
***hsa-miR-142-5p***	CATAAAGTAGAAAGCACTACT	NR_029683	chr 17q22	55
***hsa-miR-199a-5p***	CCCAGTGTTCAGACTACCTGTTC	NR_029586	chr 19p13.2	55
***hsa-miR-4454***	GGATCCGAGTCACGGCACCA	NR_039659	chr 4q32.2	55
***hsa-miRNA-181a-5p***	AACATTCAACGCTGTCGGTGAGT	NR_029611	chr 1q32.1	55
***Ce_miR39***	Quantitect Primer Assay QIAGEN (blind)	-	-	55
***RNY4***	**F**: CCGATGGTAGTGGGTTAT **R**: AAGCCAGTCAAATTTAGCA	NR_004393.1	chr 7q36.1	58
***CNP***	Hs_NPPC _2_SG QuantitectPrimerAssay QIAGEN (blind)	NM_024409	chr 2q37.1	60
***NPR-B***	**F**: ATCGCTGGCTGCTTCTAT **R**: GGTGCCTCCTTCCTGTAT	NM_002526	chr 9p13.3	60
***NPR-C***	**F**: TTCAGCATCACTCCAAGGA **R**: GTGTGGTCAGGTTAGCATA	NM_001204375	chr 5p13.3	60

**Table legend**. ***hsa-miR-33a-3p:*** homo sapiens *microRNA-33a* with 3p strand present in the reverse position; ***hsa-miR-223-5p*:** homo sapiens *microRNA-223* with 5p strand present in the forward position; ***hsa-miR-142-5p*:** homo sapiens *microRNA-142* with 5p strand present in the forward position; ***hsa-miR-199a-5p*:** homo sapiens *microRNA-199a* with 5p strand pre-sent in the forward position; ***hsa-miR-4454*:** homo sapiens *microRNA-4454*; ***hsa-miR-181a-5p*:** homo sapiens mi-croRNA-181a with 5p strand present in the forward position; ***Ce_miR39*:** *C. elegans* miR-39 miRNA mimic; ***RNY4*:** Homo sapiens RNA, Ro60-associated Y4 (RNY4), Y RNA; ***CNP*:** or *NPPC*, C-type natriuretic peptide; ***NPR-B:*** or *NPR2* or GC-B, natriuretic peptide receptor B; **NPR-C:** or *NPR3*, natriuretic peptide receptor C or clearance receptor.

## Data Availability

The data that support the findings of this study are available in IFC-CNR.
